# Multimodal In‐Sensor Computing System Using Integrated Silicon Photonic Convolutional Processor

**DOI:** 10.1002/advs.202408597

**Published:** 2024-10-28

**Authors:** Zian Xiao, Zhihao Ren, Yangyang Zhuge, Zixuan Zhang, Jingkai Zhou, Siyu Xu, Cheng Xu, Bowei Dong, Chengkuo Lee

**Affiliations:** ^1^ Department of Electrical and Computer Engineering National University of Singapore 4 Engineering Drive 3 Singapore 117583 Singapore; ^2^ Center for Intelligent Sensors and MEMS National University of Singapore 4 Engineering Drive 3 Singapore 117583 Singapore; ^3^ NUS Suzhou Research Institute (NUSRI) Suzhou Jiangsu 215123 China; ^4^ Institute of Microelectronics (IME) Agency for Science, Technology and Research (A*STAR) 2 Fusionopolis Way, Innovis #08‐02 Singapore 138634 Singapore; ^5^ NUS Graduate School‐Integrative Sciences and Engineering Programme(ISEP) National University of Singapore Singapore 119077 Singapore

**Keywords:** in‐sensor computing, multimodal sensor, photonic convolutional process, silicon photonics

## Abstract

Photonic integrated circuits offer miniaturized solutions for multimodal spectroscopic sensory systems by leveraging the simultaneous interaction of light with temperature, chemicals, and biomolecules, among others. The multimodal spectroscopic sensory data is complex and has huge data volume with high redundancy, thus requiring high communication bandwidth associated with high communication power consumption to transfer the sensory data. To circumvent this high communication cost, the photonic sensor and processor are brought into intimacy and propose a photonic multimodal in‐sensor computing system using an integrated silicon photonic convolutional processor. A microring resonator crossbar array is used as the photonic processor to implement convolutional operation with 5‐bit accuracy, validated through image edge detection tasks. Further integrating the processor with a photonic spectroscopic sensor, the in situ processing of multimodal spectroscopic sensory data is demonstrated, achieving the classification of protein species of different types and concentrations at various temperatures. A classification accuracy of 97.58% across 45 different classes is achieved. The multimodal in‐sensor computing system demonstrates the feasibility of integrating photonic processors and photonic sensors to enhance the data processing capability of photonic devices at the edge.

## Graphical abstract

The work demonstrates a photonic multimodal in‐sensor computing system by combining a photonic sensor and a photonic processor. By integrating the photonic sensor with a photonic processor performing convolutional operation, the complex spectroscopic data is convolved for classification with a high accuracy, proving the feasibility of photonic in‐sensor computing.

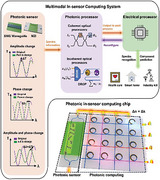

## 1 Introduction

The increasing demand for artificial intelligence of things (AIoT) and cloud computing have spurred extensive research into modern intelligent hardware systems for data processing.^[^ ^1^, ^2^, ^3^, ^4^ ^]^ The number of sensor nodes in the AIoT is projected to reach 125 billion by 2030.^[^ ^5^ ^]^ Multimodality is a desired property at each sensor node to provide comprehensive sensory information. Multimodality sensor further increases the data volume generated at each sensor node. Traditional AIoT systems require the transfer of all sensory data, including a huge redundancy, to the cloud computing platform for data processing.^[^ ^6^, ^7^ ^]^ This approach causes high communication bandwidth consumption, high power consumption, and high network latency.^[^ ^8^, ^9^, ^10^ ^]^ To address these challenges, in‐sensor computing and near‐sensor computing have been demonstrated to transfer some computing tasks from the cloud to edge devices to ease the burden on communication channels.^[^ ^11^, ^12^, ^13^, ^14^, ^15^ ^]^ Recently, a few studies have reported neuromorphic devices that integrate multimodal sensing functions into a single device.^[^ ^16^, ^17^, ^18^, ^19^, ^20^ ^]^ However, these devices have limited sensing capabilities, with most only able to detect two types of sensor information.^[^ ^17^, ^21^ ^]^ Maintaining simultaneous input data fusion is crucial for multimodal in‐sensor computing systems.^[^ ^22^ ^]^ Despite that most in‐sensor and near‐sensor computing platforms are based on electronics,^[^ ^23^, ^24^, ^26^, ^27^, ^28^ ^]^ photonics is an alternative route for in‐sensor and near‐sensor computing which provides high speed, high sensitivity and high energy efficiency.^[^ ^29^, ^30^, ^31^, ^32^, ^33^ ^]^ To exemplify this, photonic sensors have been applied in AIoT systems for complex gas and liquid sensing, capable of detecting more than three different chemicals.^[^ ^34^, ^35^, ^36^, ^37^ ^]^ On the one hand, photonic multimodal sensors inherently possess the advantage of simultaneously fusing multiple sensory signals into one spectrum.^[^ ^38^, ^39^, ^40^, ^41^, ^42^ ^]^ On the other hand, photonic processors have been advancing rapidly in the past decade for computations in artificial intelligence, a bedrock for processing complex photonic spectroscopic sensory data. It is thus natural to seek advancements to combine photonic sensors and photonic processors toward photonic in‐sensor computing.

**Figure 1:**
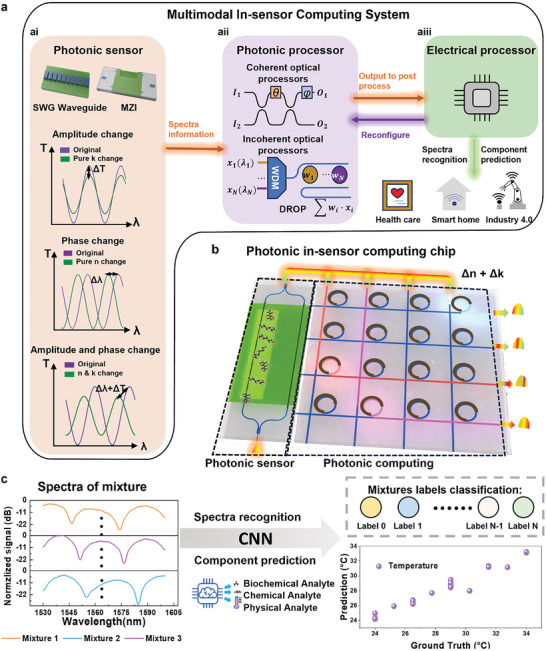
Overview of our multimodal in‐sensor computing system. a) Workflow of the multimodal in‐sensor computing system. The guided‐wave sensor obtains spectral information and passes it to the photonic processor. After data processing, the configured photonic processor then transmits the outputs to electrical post‐processing for further spectra recognition and component prediction. ai) Basic configurations of photonic sensors. The schematic of the sensing mechanisms of photonic sensors can be divided into two parts: those based on amplitude changes, such as straight fibre waveguides and subwavelength grating (SWG) waveguides, and those based on phase shifts, such as microring resonators and Mach‐Zehnder interferometers. aii) Photonic processors can generally be divided into two parts: one based on coherent processes and the other on non‐coherent processes. aiii) The electrical processor completes the remaining processing tasks, performs spectral recognition, and reconfigures the photonic computing system accordingly. b) The multimodal photonic sensors transfer the spectra information to photonic processor for further data process. c) Machine learning for mixtures spectra classification and component prediction using convolutional neural network.

Photonic sensors have two typical categories. One is phase‐based sensors such as Mach‐Zehnder interferometers (MZIs) and microring resonators (MRRs). The other is absorption‐based sensors such as channel waveguides, slot waveguides, and subwavelength grating (SWG) waveguides.^[^ ^43^, ^44^, ^45^ ^]^ Phase‐based sensors rely on the change of the real part (*n*) of refractive index (RI) which causes the wavelength shift. Absorption‐based sensors make use of the amplitude change induced by the change of the imaginary part (*k*) of RI, as shown in **Figure** **1**a–i.^[^ ^46^, ^47^, ^48^ ^]^ Most photonic sensors only use either *n* or *k*, resulting in limited sensory information which is not capable of supporting multimodal sensing desired in complex environments at the edge. MZI sensors have the potential for multimodal sensing by simultaneously utilizing both *n* and *k* together with spectroscopic information. The simultaneous change of *n* and *k* causes dip wavelength change and transmission change across a broad spectrum, making MZI sensors capable of capturing various stimuli information. This approach can fuse multiple analytes information into one spectrum, reducing the footprint of the entire sensor system by removing the need for multiple sensors for multiple analytes. However, while reducing the footprint and simplifying hardware, the compromise lies in the increased complexity of multimodal sensory information processing. Convolutional neural networks (CNN) can help address such complex problems with their advanced data analysis capabilities.^[^ ^49^, ^50^ ^]^

Convolutional processing can be implemented by photonic processors.^[^ ^51^, ^52^, ^53^, ^54^, ^55^, ^56^, ^57^, ^58^ ^]^ By leveraging the unique advantages of light, photonic processors can achieve superior computational capabilities because of their ultra‐low power consumption and powerful parallel data processing capabilities. Several photonic processor architectures have been reported in current research, and they can generally be classified into coherent and incoherent types as shown in Figure 1a–ii. Coherent photonic processors are mainly based on MZIs and diffractive spatial light networks, while incoherent photonic processors include wavelength division multiplexing(WDM) crossbar arrays or based on fibre dispersion and phase change material crossbar arrays.^[^ ^59^, ^60^, ^61^, ^62^, ^63^, ^64^ ^]^ MRR weight bank is also a mainstream architecture.^[^ ^65^, ^66^, ^67^, ^68^, ^69^ ^]^ The MRR weight bank features favourable scalability by utilizing WDM, and its wavelength‐selective nature is well‐suited for spectroscopic data processing.^[^ ^70^, ^71^, ^72^ ^]^

Here, we demonstrate an in‐sensor computing system by combining a photonic multimodal spectroscopic sensor and a photonic processor (Figure 1a). The MZI multimodal sensor makes full use of the *n* and *k* and transmits spectroscopic information of two kinds of proteins of different concentrations and temperature conditions to the photonic processor. The photonic processor with 5‐bit accuracy does convolutional operation at the edge (Figure 1b). Then, the output from the photonic processor is then sent to the cloud, where electrical post‐processing performs spectral recognition of mixtures with 45 mixing ratios using a convolution neural network (CNN) (Figure 1a–iii). This approach has a classification accuracy of 97.85% and is able to predict component concentration as shown in Figure 1c. Our proposed multimodal in‐sensor computing system provides a low‐power and efficient solution by combining spectroscopic waveguide sensing technique with photonic processor which paves a new way toward future AIoT sensing systems, including health care, smart home, and industry 4.0.

## 2 System Design and Characterization

### 2.1 The Principle of Photonic Multimodal In‐Sensor Computing System

Conventional photonic sensors only analyze either the wavelength or the amplitude change, which is easily influenced by the external environment.^[^ ^73^, ^74^, ^75^, ^76^ ^]^ Such a single sensing mechanism encountered difficulties in the multimodal sensing application, which is usually used in single‐component sensing. There are also spectroscopic sensors that utilize the absorption of different molecules for multimodal sensing just use *k*.^[^ ^77^, ^78^, ^79^, ^80^, ^81^, ^82^ ^]^ For a multimodal MZI sensor, there is potential for multi chemical/biochemical mixture sensing by utilizing changes in both *n* and *k*.^[^ ^83^, ^84^ ^]^ When the *k* changes in one sensing arm, the extinction ratio (ER) alters. When the *n* changes in one sensing arm, the dip wavelength shifts. However, when both *n* and *k* change simultaneously, analysis becomes challenging due to complex overlapping, especially when spectroscopic information is taken into account.

The unique molecular fingerprints enable photonic MZI sensors to detect various analytes by measuring changes in ER and dip wavelength shifts, providing specific identification of chemicals and biomolecules due to their different *n* and *k*. In practical sensing environments, mixture sensing and analysis are crucial for many applications. While specific molecular fingerprints can identify different chemicals and biochemicals, quantitative and qualitative analysis of chemical and biochemical mixtures remains challenging due to the overlapping absorption spectra of substances with similar chemical bonds. Furthermore, the temperature perturbations also lead to changes in the effective RI, which increases the difficulty of analysis. With the rapid advance of machine learning in recent years, many research fields have utilized this technology to harness its advanced data analysis capabilities. Using CNN, it is possible to recognize the spectra of mixtures with predefined mixing ratios and implement component prediction. The integrated silicon photonic convolutional processor (ISPCP) in our multimodal in‐sensor computing system is able to perform convolutional processing.

In our proof‐of‐concept multimodal in‐sensor computing system, the spectra information is first collected from the photonic sensor for neural network training. An ex‐situ machine learning method is used to train CNN kernel weights, which are then deployed on the ISPCP. After configuration, performing computations at the edge of the photonic sensing terminal reduces the redundant data transmitted between the sensing terminal and the processing units. Next, for new spectra information, the ISPCP can process data to replace the processing at the cloud. Then the output from the ISPCP is transmitted to the electrical post‐processing. This approach enables in‐sensor computing of photonic multimodal signals, significantly reducing redundancy and power consumption associated with machine learning tasks.

In our in‐sensor computing system, an MRR crossbar array is used as ISPCP which offers several advantages. MRR crossbar arrays are compact and easily integrated, enabling accurate and complicated calculations after the calibration process. MRR crossbar array schemes also show great advantages in small‐size and large‐scale applications, satisfying the demands of current edge computing for photonic sensor device research.

To leverage the MRR crossbar array, four laser wavelengths are used in our multimodal in‐sensor computing system. For spectrum information processing, the input spectrum from the sensor was initially reshaped into a 1D tensor (Figure S1, Supporting Information). Then every four elements of the tensor are loaded onto the variable optical attenuators (VOAs) to achieve parallel processing. After passing through the ISPCP, the data was collected by photodetectors (PDs) where the optical signal is converted to electrical signal. Peripheral circuits were used to minimize noise and amplify the signal from PD. Each PD readout represents one kernel result.

### 2.2 The Characterization of ISPCP

**Figure 2:**
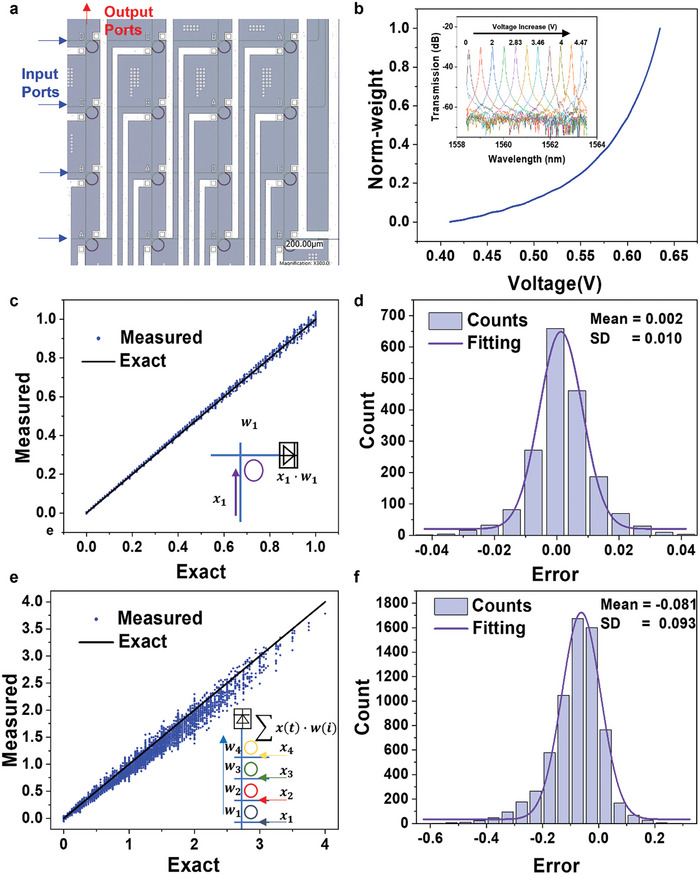
The characterization of ISPCP. a) The micrograph of the MRR array. b) The normalized W‐V mapping of one MRR on the chip. The insert shows the drop spectra of a single MRR under different power tuning (0–30 mW, 3.6 mW step^−1^). c) Measured scalar multiplication results using a single MRR versus exact results. d). A histogram of computational error calculated by subtracting the measured scalar multiplication results from the exact results. The histogram is fitted by a Gaussian distribution (red solid curve). SD standard deviation. e) Measured multiply‐accumulate results using a 4*1 MRR array versus exact results. f) A histogram of computational error calculated by subtracting the measured multiply‐accumulate results from the exact results. The histogram is fitted by a Gaussian distribution (red solid curve).

**Figure** **2**a shows the ISPCP chip, fabricated by a silicon‐on‐insulator (SOI) integration process at Advanced Micro Foundry (AMF). This chip is compact, containing a 4 × 4 MRR array consuming an area of 0.64 mm × 0.64 mm. This 4 × 4 MRR array forms the core of the in‐sensor computing system that takes charge of computing. The MRR device is the basic computational unit and is tuned by titanium nitride (TiN) heaters because of the high thermo‐optic coefficient of Si. Figure S2 (Supporting Information) shows an optical microscopic image of one MRR in ISPCP, showcasing the intricate details of a single MRR. When the TiN heater is heated through the voltage applied, the resonant condition changes, which allows precise manipulation of the resonance wavelength. Meticulous heater design considerations have been incorporated to ensure accurate voltage control of the MRR. Specifically designed peripheral electronic circuits and a control unit are used to precisely control the ISPCP. A 12‐bit resolution digital‐to‐analogue converter (DAC) with multiple outputs is programmed to apply the voltage. The chip is affixed on a thermo‐electric cooler (TEC) module through the thermal adhesive to keep the stability of the measurement setup as shown in Figure S3 (Supporting Information). The TEC module plays an important role in the precise control of the ISPCP to reduce the thermal crosstalk when adjusting multiple MRRs simultaneously. The fibre array is used as the optical signal input/output (I/O) connection. To facilitate efficient electrical I/O connections, the ISPCP chip is wire bonding to a well‐designed PCB.

Similarly to other crossbar array schemes, the look‐up tables of the VOAs and MRR array are established first to obtain the weight‐voltage (W‐V) mapping. During the MRR look‐up table establishment, we scanned the MRR spectrum first. Then, the wavelength of laser is chosen near the resonance wavelength of each MRR to minimize the noise and improve accuracy. The applied voltage of the VOA is fixed, and the voltage applied to the MRR is incrementally adjusted in a step of 2.4 mV (10*V*/2^12^). The optical power of the drop port is acquired by a 12‐bit ADC to collect the PD output voltage. After normalization, a W‐V curve is established by the control unit that describes the relationship between weight and voltage applied to MRR. Figure 2b illustrates the W‐V curve from one MRR of the ISPCP chip. The inset shows that increasing the voltage on the MRR leads to a redshift in the resonance wavelength. Figure S4 (Supporting Information) shows the relationship between power consumption and the peak wavelength shift. To examine the thermal crosstalk of the ISPCP, one ring is heated, while the rings nearby are measured as shown in Figure S5a (Supporting Information). When one MRR is heating, the resonant wavelength of the nearby MRRs remain almost unchanged as shown in Figure S5b (Supporting Information). However, the zoom‐in picture in Figure S5c (Supporting Information) shows that there is still a little change in resonant peak wavelength, which causes the accuracy of multiply‐accumulate (MAC) to decrease compared to a single ring. Due to fabrication variations, the spectrum of each MRR differs, requiring a unique look‐up table for each. Further details are provided in Note S4 (Supporting Information). For VOA W‐V curve establishment, the laser operating wavelength is chosen the same as the MRR working wavelength. The MRR tuning voltage is kept constant while the VOA applied voltage is adjusted. Throughout this process, the optical power of the drop port is continuously detected. After normalization, a W‐V curve is established that describes the relationship between normalized input data and applied voltage on VOA shown in Figure S7 (Supporting Information).

After getting the MRR and VOA look‐up tables, we demonstrated scalar multiplication on one single MRR with almost 6.6‐bit accuracy. The applied voltage on VOA is zero, which means the normalized data through the VOA is 1. And 1320 random numbers from 0 to 1 are chosen to demonstrate the scalar multiplication. Figure 2c shows a good matching between the exact and measured results of a single MRR multiplicatio. The standard deviation (SD) of the residual error is as low as 0.010, which shows the computing accuracy is 6.6‐bit, and the mean error is −0.002 fitted by a Gaussian distribution as shown in Figure 2d.

After single MRR multiplication was examined, the MAC operation was also examined. The multiplication is done by MRRs, while the accumulation is done by PD. First, four different wavelengths are chosen for the one patch. Then the W‐V look‐up tables of the 4 MRRs in one patch were established. The voltage applied to VOA is zero, which means the input data(*x*_1_,*x*_2_,*x*_3_,*x*_4_) is normalized to 1. Figure 2e shows a good matching between the exact and measured results of a 1*4 convolution result. The standard deviation (SD) of the residual error is 0.093, and the mean error is −0.081 fitted by a Gaussian distribution, which means ISPCP can do a 5.4‐bit MAC operation as shown in Figure 2f. Such accuracy degradation from one MRR to four MRRs is caused by thermal crosstalk. There are many methods to improve the computational precision of the ISPCP chip, such as a dual‐wavelength self‐calibration process,^[^ ^85^ ^]^ building a thermal cross‐talk model,^[^ ^86^ ^]^ using feedback weight control methods,^[^ ^87^ ^]^ using a dithering control scheme.^[^ ^88^, ^89^ ^]^

### 2.3 Operation for Convolution and Edge Detection

**Figure 3:**
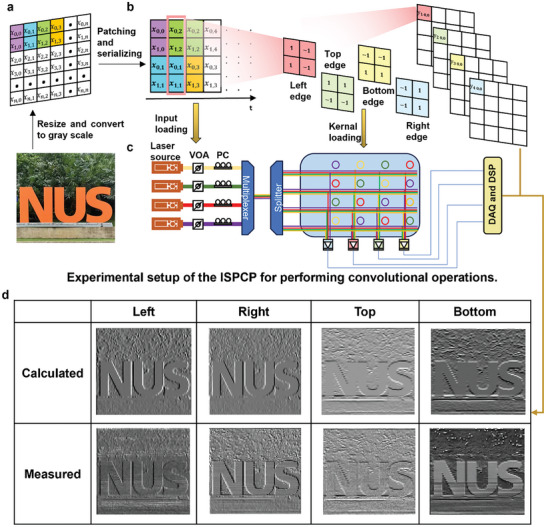
Convolutional operation using ISPCP. a) A test picture for image edge detection experiment. Data matrix of a grey image (95 × 83) and original image used for demonstrating the convolution effect. b) Edge convolution kernels used. c) Experimental setup of the ISPCP for performing convolutional operations. d) Corresponding convolution image results.

To examine the convolutional operation of the ISPCP chip, the chip is programmed to perform edge detection of images by loading convolutional kernel matrices. The information data are transmitted and encoded in the optical power. Four 2 × 2 image kernels are used to do convolution of an input image, processing one 4 × 1 vector per time step on ISPCP chip. **Figure** **3**a shows the original image used in the edge detection test, a 95 × 83 pixel image of the NUS logo. The input image was initially resized to 95 × 83 pixels and converted to grayscale. The image was then flattened into a 2D vector with 4 rows, as shown in Figure 3b. Then, every four elements in one column were grouped and loaded onto the VOAs. Once the data is serialized, it flows toward the ISPCP chip, which works as the convolution processor. The kernel matrix $\left[\begin{array}{cc} -1 & 1\\ -1 & 1 \end{array}\right]$ is for right edge detection($\left[\begin{array}{cc} 1 & 1\\ -1 & -1 \end{array}\right]$ for top, $\left[\begin{array}{cc} 1 & -1\\ 1 & -1 \end{array}\right]$ for left, and $\left[\begin{array}{cc} -1 & -1\\ 1 & 1 \end{array}\right]$ for bottom edges). To present the negative values, the offset matrix is used, which is discussed in reference.^[^ ^59^ ^]^ Within the ISPCP chip, each ring was tuned to a convolutional kernel weight according to the W‐V curve shown in Figure 3c. The input values underwent MAC operations along the row. Ultimately, the results of the convolutional operations were acquired by a PD for further processing. Figure 3d visually presents the outcome of four edge detection operations through ISPCP chip. The experimental results provided substantial evidence supporting the feasibility of utilizing the ISPCP chip within our in‐sensor computing system for convolutional computing.

### 2.4 Photonic MZI Sensor Single Analyte Measurement

**Figure 4:**
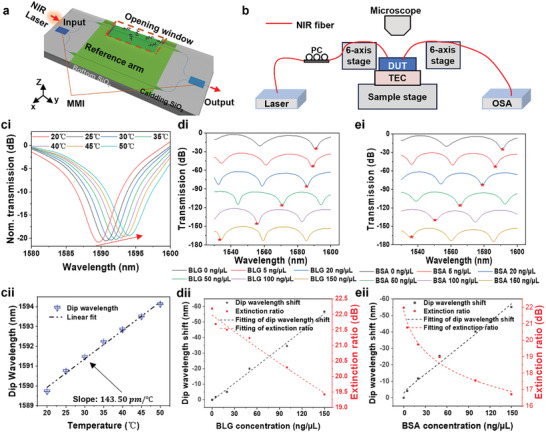
Measurement for single analyte. a) Detailed view of the proposed sensor structure. b) Schematic of the photonic sensing testing setup. ci) Measured spectra of test devices for seven different temperatures from 20 to 50 °C. cii) The shift of the dip wavelength as a function of the temperature. di‐ ei) Transmission spectra evolution at wavelengths ranging from 1530 to 1600 nm for BSA and BLG concentration change. dii‐eii) Dip wavelength shift and extinction ratio change at differenet BSA and BLG concentrations and their fitting.

An unbalanced MZI modulator was designed as a multimodality sensor, as shown in **Figure** **4**a. The opening window was 700 µm on one arm, and the unbalanced MZM had a path difference of 30 µm between the two arms. A slab waveguide was used as the arms of MZI. Figure S8 (Supporting Information) shows the 1D relative intensity profile along the x‐direction at z = 0. The sensing performance of our platform was characterized by a custom‐built setup depicted in Figure 4b. In this setup, a C‐band laser (Keysight 81960A) was swept over the sensor resonance. After passing through the photonic device, the laser was detected by a large sensing area power meter (Keysight 81636B). The light was coupled into and out of the MZI sensor using on‐surface focusing grating couplers. As shown in Figure 4c–i, the evolution of the optical spectral response of the MZI sensor was observed when the temperature varied from 20 to 50 °C. The wavelength shifted toward longer wavelengths with increasing temperature, starting from 1589.2 nm. Figure 4c–ii shows the dip wavelength shift concerning temperature. This multimodal sensor have the sensitivity of 143.5 *pm* °C for temperature sensing.

The thin‐film sensing process for β‐lactoglobulin (BLG) proteins and bovine serum albumin (BSA) is demonstrated. BLG is a major protein in milk, while BSA is a major protein in bovine serum. We tested samples of BLG and BSA prepared at concentrations ranging from 0 to 150 ng µL^−1^ in DI water. A 0.2 µL protein solution was dropped onto the sample to form a thin film which can change both the real and imaginary part of refractive index of the sensing arm, which causes the wavelength shifts and ER change in the spectrum. To quantify the thickness of the film on the sensor device, we use AFM (Bruker Dimension FastScan) to measure the surface microscopy image. We dropped 0.2 µL of 100 ng µL^−1^ BSA solution onto the sample. After the solution was dried, we took the AFM image (Figure S9, Supporting Information). The BLG is also measured. Due to differences in concentration, the thickness of the thin film varied. The simulation results between the film thickness and *n,k* are shown in Figure S10 (Supporting Information). The thickness difference caused variations in propagation loss and effective RI of sensing arm. The spectra with different protein concentrations are plotted in Figure 4d–i,e–i. We further plotted the dip wavelength change and extinction ratio change with concentrations in Figure 4d–ii,e–ii along with their fittings, corresponding to our simulation in Figure S10 (Supporting Information). And the relationship between loss and extinction ratio is shown in Equation S9 (Supporting Information). For refractive index sensing mechanism, the sensitivity for BLG and BSA are − 0.360 nm/(ng/µL) and − 0.374 nm/(ng/µL). The detection limit are 2.77 × 10^−4^ ng/µL and 2.67 × 10^−4^ ng/µL. For absorption sensing mechanism, the sensitivity of BLG and BSA are − 0.0168 *dB*/(ng/µL) and − 0.031 *dB*/(ng/µL) extrated from the slope of the curve. The detection limit of BLG and BSA are 2.39 × 10^−3^ ng/µL and 2.41 × 10^−3^ ng/µL drived by 3‐sigma rule and noise measurement (Figure S12, Supporting Information).

### 2.5 Convolutional Neural Network for Recognition of Protein Mixture Absorption Spectrum Using ISPCP Chip

In recent years, machine learning has demonstrated its capability to handle complex data for tasks such as feature extraction and image classification. Among various algorithms, CNNs have shown excellent performance in sensing signal classification. We also experimented with Multilayer Perceptrons (MLPs) and CNNs for classification, as shown in Figure S13 (Supporting Information). The simulation shows CNNs have better performance than MLP in spectra classification tasks.

**Figure 5:**
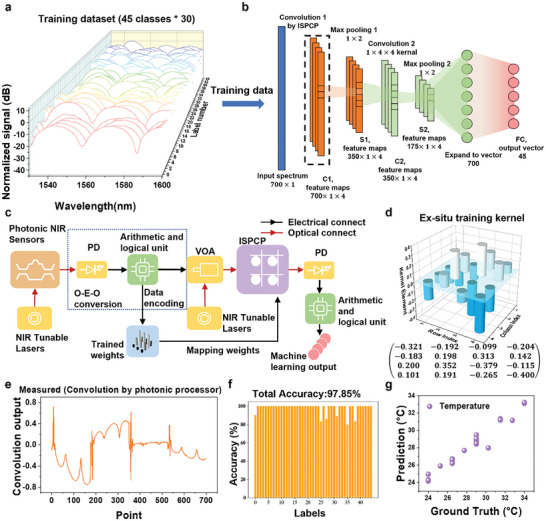
Spectra classification and component prediction using ISPCP. a) The visualization of a portion of mixture spectra in the training data set. The training data set consisted of 45 concentration combinations, each having 30 spectra. b) The detailed architecture of the CNN training model. The first convolution kernel is processed by ISPCP. c) The data flow chart of in‐sensor computing system. d) The kernel generated by ex‐situ training. e) An example of the convolution output of the ISPCP chip. f) The bar charts of testing accuracy in identifying 45 mixtures through ISPCP. The classification accuracy is 97.85%. g) Prediction of temperature.

We employed CNNs to differentiate 45 different mixture spectra. **Figure** **5**a shows the mixture spectra data after normalization, which will be fed into the CNN for classification. The correspondance between mixtures of different biomolecule types, concentrations, and temperatures and labels 0–44 is shwon in Table S1 (Supporting Information). Each spectrum consists of 700 wavelength points, ranging from 1530 to 1600 nm, with an interval of 0.1 nm. By mapping the 45 mixing ratios to labels from 0 to 44, the input mixture spectrum is classified into specific labels representing the mixing ratio.

Figure 5b depicts the detailed 1D‐CNN structure that we employed for the classification task. For data division, 30 spectra in each class were split with a 4:1 ratio, using 24 for training and 6 for testing. The accuracy of the testing set data for the 45 classes of the gas mixture reached 98%, as shown in Figure S14 (Supporting Information), demonstrating the strong capability and stable sensing performance of our proposed waveguide sensing platform.

Next, we used the ISPCP chip to perform the first layer convolution. In the ex‐situ trained CNN, four convolutional kernels form the first layer, each with a size of 1 × 4. The first layer of convolutional operations was performed entirely by the ISPCP chip. Like the previous image edge detection experiments, the input spectrum data were reshaped into four rows and flowed into the ISPCP chip, where they were convolved with the pertained kernels. After the first convolutional layer, the output size became 700 × 1× 4. Followed by one convolutional layer,  two max‐pooling layers, and fully connected layers were applied which are consistent with the previous fixed layers trained on electrical post‐process.

The detailed operation principle in terms of information conversion and information flow is illustrated in Figure 5c. The environmental information is first collected by the NIR multimodal MZI sensor and encoded as spectrum data in the optical domain. This data is then collected by a PD, which converts the spectrum information into electrical signals and transmits it to a computer, where optical‐to‐electrical conversion occurs. The computer initially performs deep learning on the data, training the model and obtaining the fixed layers. The resulting weights are then transferred to the ISPCP. For new data, the computer resizes the spectrum information and converts it to optical information using the VOA as mentioned earlier. The outputs of the ISPCP are collected and further transmitted to the computer for further processing.

The training method of the ISPCP in‐sensor computing system includes two stages. First, a CNN model is trained ex‐situ. The elements of the first convolution layer after training are shown in Figure 5d. Then all the determined weights are transferred to the ISPCP chip using the look‐up table method. One of the convolution outputs from ISPCP is shown in Figure 5e. Then the convolution output from the ISPCP chip is transmitted to the trained CNN model where the first convolution layer is deleted. The accuracy of the CNN task performed by the ISPCP is 97.85%, as shown in Figure 5f. Notably, for electronic computation, the model achieves a recognition accuracy of 98% in Figure S14 (Supporting Information). The slightly lower accuracy of the CNN on the ISPCP compared to the PC is due to the precision limitations of the ISPCP chip.

Although the CNN outcome presents a high accuracy, the component concentration information of each spectrum is still missing. To get the prediction of the component concentrations, we modified the final layer of such CNN to make it output the component's concentration. The whole network is retrained for the prediction task. The prediction of temperature is shown in Figure 5g. The other prediction results of the other two kinds of protein are shown in Figure S15 (Supporting Information). The estimated power efficiency of our multimodal in‐sensor computing system is ≈2.9 *TOPS*/*W*, and the detailed calculation is shown in Note S11, Supporting Information). To complete this machine learning task, our multimodal in‐sensor computing system is 30 times more efficient than current GPUs. The detailed calculations are provided in Note S13 (Supporting Information).^[^ ^90^ ^]^ These two tasks indicate that the ISPCP chip enables accurate classification tasks and prediction tasks for multimodal spectroscopic sensors.

## 3 Conclusion

The rapid development of the AIoT and cloud enlarges the communication cost between the sensor and data process unit. The multimodal sensing requirements increase the data volume, including redundancy, which increases the analysis difficulty, necessitating an in‐sensor computing system to decrease the redundancy. Photonics is an alternative route for in‐sensor and near‐sensor computing with high sensitivity, high speed and high integration level based on a mature CMOS manufacturing process.

Photonic sensors and photonic processors have been advanced in the past decade for their deployment in AIoT systems and machine learning processing systems. But they were advanced independently. Combining these two technologies is the natural next‐step, as they share the same fundamental optics physics and manufacturing platform. More importantly, such a combination is not simply an addition. By synergizing photonic sensors and photonic processors, photonic in‐sensor computing systems can be constructed, which address the huge redundant data communication issue in AIoT systems based on cloud communication framework.

Our work precisely demonstrates such a photonic multimodal in‐sensor computing system by combining a photonic sensor and a photonic processor. The photonic sensor utilizes the changes in both the real part and imaginary part of refractive index to harness complex spectroscopic data which can carry comprehensive multimodal sensory information beyond the capability of the sole use of real part or imaginary part. By integrating the photonic sensor with a photonic processor capable of performing convolutional operation at 5‐bit accuracy, the complex spectroscopic data is convolved for CNN classification. In the task of classifying 45 different combinations of biomolecule types, concentrations, and temperatures, we obtain classification accuracy almost identical to that of traditional electronic computation, achieving an accuracy of 97.85% with the aid of well‐designed peripheral circuits and control algorithms. The exceptional performance in image edge detection and convolution tasks opens new possibilities for other edge computing applications. Such high accuracy proves the feasibility and effectiveness of photonic in‐sensor computing, which is essential in reducing data redundancy in future large‐scale expansion AIoT systems.

Looking forward, with the rapid development of the photonic circuit, more functions required in our in‐sensor computing system can be integrated on‐chip, though these functions are currently off‐chip. For example, the activation function can be integrated on the same chip, which will reduce the power consumption and communication latency of our in‐sensor computing system.^[^ ^91^, ^92^ ^]^ Tunable laser and photonic memory can also be integrated.^[^ ^93^, ^94^, ^95^ ^]^ The photonic ADC/DAC can further improve the whole system's performance.^[^ ^96^, ^97^ ^]^ Our multimodal in‐sensor computing system offers an effective solution for complicated, multi‐scenario AIoT tasks in healthcare, smart home and industry 4.0 applications.

## 4 Experimental Section

### Characterization of the MZI Sensor

For the optical characterization, the light was emitted from a tunable laser source covering 1530 to 1600 nm with a minimum tuning step of 10 pm (Keysight 81960A Tunable Laser). The light was guided through a single‐mode‐maintaining polarization controller (THORLABS, FPC562), focused on the grating coupler by a single fiber (THORLABS, P1‐SMF28E‐FC‐1), and finally directed to the MZI sensor. The optical output spectrum from the MZI sensor was measured by a power meter (Keysight 81636B Power Sensor) which was synchronized with the tunable laser source.

### Characterization of the ISPCP

For the optical characterization, the light was emitted from a tunable laser source (SIMTRUM's C‐Band Tunable Laser). The light was guided through a polarization controller, and then going through a MUX and a splitter. Then the light is focused on the grating coupler by a fiber array (PLC CONNECTIONS, 12‐Ch SMF‐28 Fiber Array for Grating Coupler), and finally directed to the ISPCP. The optical output spectrum from the ISPCP was measured by a biased detector (THORLABS, DET08CFC/M). For the electrical characterization, the MCU controller (STMicroelectronics, NUCLEO‐F746ZG) was used to control the 12‐bit ADC board (MAX22531EVKIT) to collect the analogue output from the amplifying circuit after the photodetector. The MCU controller also controls the DAC board with 12‐bit accuracy (MAX11300PMB1) to apply the voltage. The MCU controller was connected to the PC via USB cable communication for instruction and information transmission. The frequency of the whole system is limited by the speeds of DAC, which operates at 1kHz.

## Conflict of Interest

The authors declare no conflict of interest.

## Supporting information



Supporting Information

## Data Availability

The data that support the findings of this study are available from the corresponding author upon reasonable request.
