# Supplementary material for: Multimodal In‐Sensor Computing System Using Integrated Silicon Photonic Convolutional Processor
Source: Adv Sci (Weinh). 2024 Oct 28;11(47):2408597. doi: 10.1002/advs.202408597 (PMC11653661; doi:10.1002/advs.202408597)
Supplement: Supplementary file 1 — Supporting Information [file ADVS-11-2408597-s001.docx]

Supporting Information

Multimodal In-sensor Computing System Using Integrated Silicon Photonic Convolutional Processor

*Zian Xiao, Zhihao Ren, Yangyang Zhuge, Zixuan Zhang, Jingkai Zhou, Siyu Xu, Cheng Xu, Bowei Dong*, and Chengkuo Lee**

Contents

Note S1 Operation principle of integrated silicon photonic processor for convolutional operations. 2

Note S2 Single microring resonator device and packaged layout chip 3

Note S3 Power consumption of single microring and the crosstalk in array 5

Note S4 Storage of look-up table 7

Note S5 Variable optical attenuator characterizes 9

Note S6 Theoretical analysis and fitting of Mach-Zehnder interferometer sensor 10

Note S7 Calculation of limit of detection (LoD) of Mach-Zehnder interferometer sensor 15

Note S8 Comparison of MLP and CNN for spectrum classification 17

Note S9 The bar charts of testing accuracy on PC 19

Note S10 The prediction of BSA and BLG 20

Note S11 Power consumption of whole system estimation 21

Note S12 Comparison with other sensing system. 22

## Note S1 Operation principle of integrated silicon photonic processor for convolutional operations.


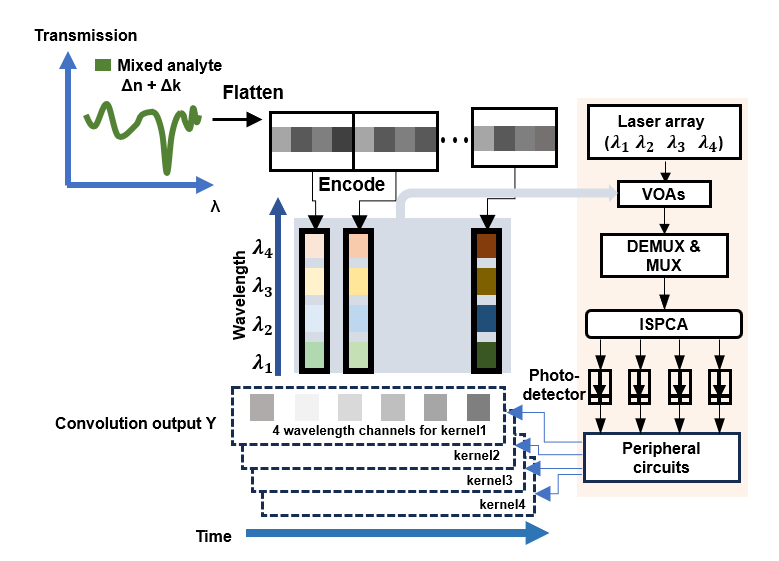


**Figure S1.** Operation principle of integrated silicon photonic convolutional processor (ISPCP). The variable optical attenuators (VOAs) are used to encode the laser intensity.

The input data was flattened into one-dimension vector, and then encoded through VOAs in optical intensity with 4 wavelengths. The photodetectors (PDs) get the results of convolution operation in optical intensity through ISPCP. The peripheral circuit help to enhance the output of the photodetector and lower the electrical signal noise. Each PD get the convolution output of one kernel.

## Note S2 Single microring resonator device and packaged layout chip


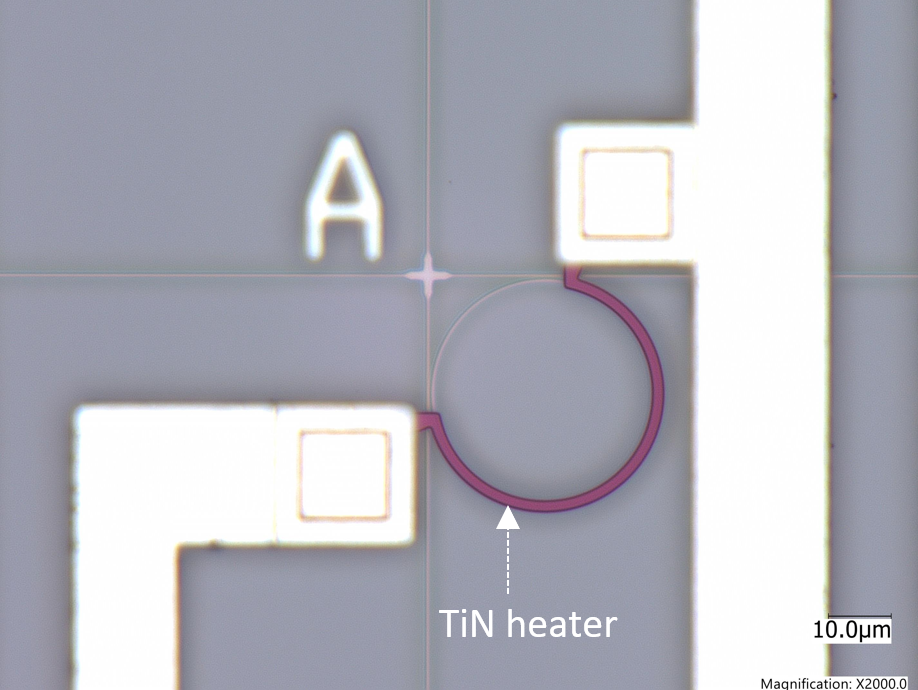


**Figure S2.** Optical microscopic images of a single ring in the array. The radius of the MRR in the picture is 17.99μm, and the gap between ring and bus waveguide is 300nm. The waveguide width is 500nm. The waveguide crossing uses the Advanced Micro Foundry (AMF) standard cell.


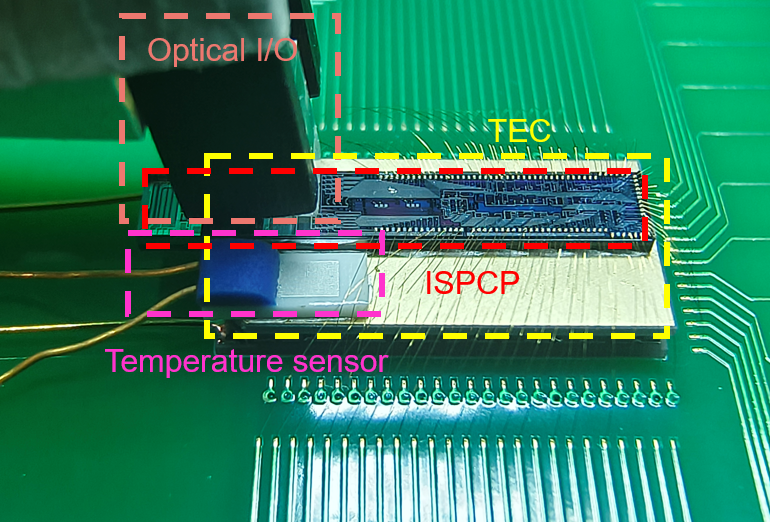


**Figure S3.** Detailed photos of the packaged layout chip show the MRR array on a thermoelectric cooler (TEC) (BITE-SUPPER, MTE22Z0301), with the photonics chip wire bonded to the customized printed circuit board (PCB) for computation and control. On the left, there is an optical input/output port using a 12 ports fiber array, and the entire assembly is mounted on a TEC with temperature sensor (THORLABS, TH100PT) for PID controller (THORLABS, TED4015) for heat dissipation.

## Note S3 Power consumption of single microring and the crosstalk in array

**Figure S4.** Linear fit of the single microring resonant wavelength shifts at different power consumption.

The resonant wavelength shift is linear to the power consumption, so, the work wavelength can be chosen close to resonant wavelength to minimize the power consumption of whole system.


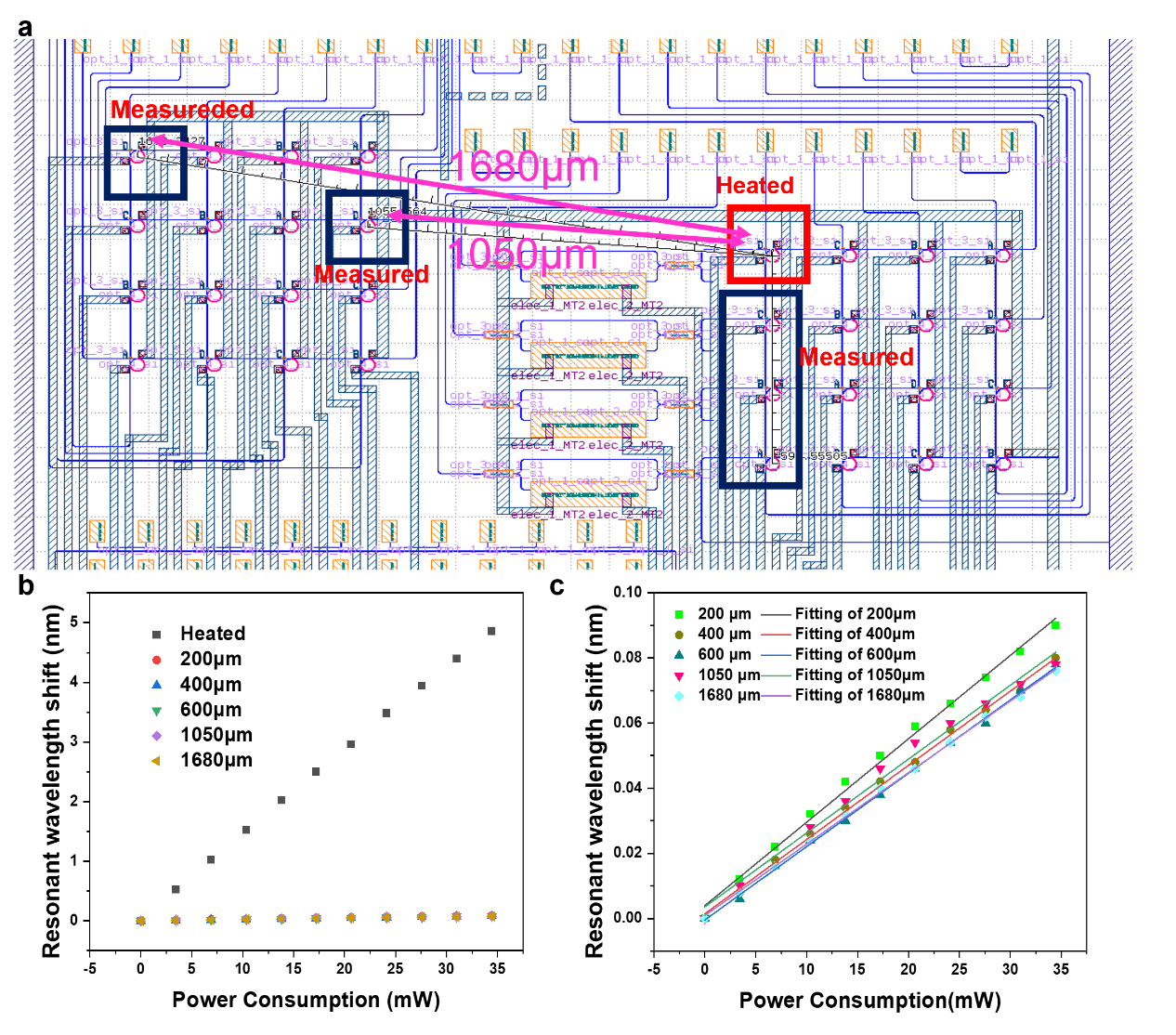


**Figure S5.** Thermal crosstalk of the MRR array. (a) Layout of the heated ring and measured rings. (b) Resonant wavelength shifts of heated ring and measured rings. (c) Zoom-in wavelength shifts of measured rings.

To examine the crosstalk in the array, one microring is heated while the neighboring microrings are measured. In **Figure S4a**, the ring within the red box is heated, and the rings within the black boxes are measured. The distances between the heated device and the measured devices are 200μm, 400μm, 600μm, 1050μm, and 1680μm. **Figure S4b** indicates the wavelength shifts in the other rings. However, small wavelength shifts in the other rings are evident in the zoomed-in view shown in **Figure S5c**. The slope of the resonant wavelength shift for the ring distances from 200μm to1680μm shows almost no change, indicating that the crosstalk between the heated ring and the others is consistent across this range.

## Note S4 Storage of look-up table


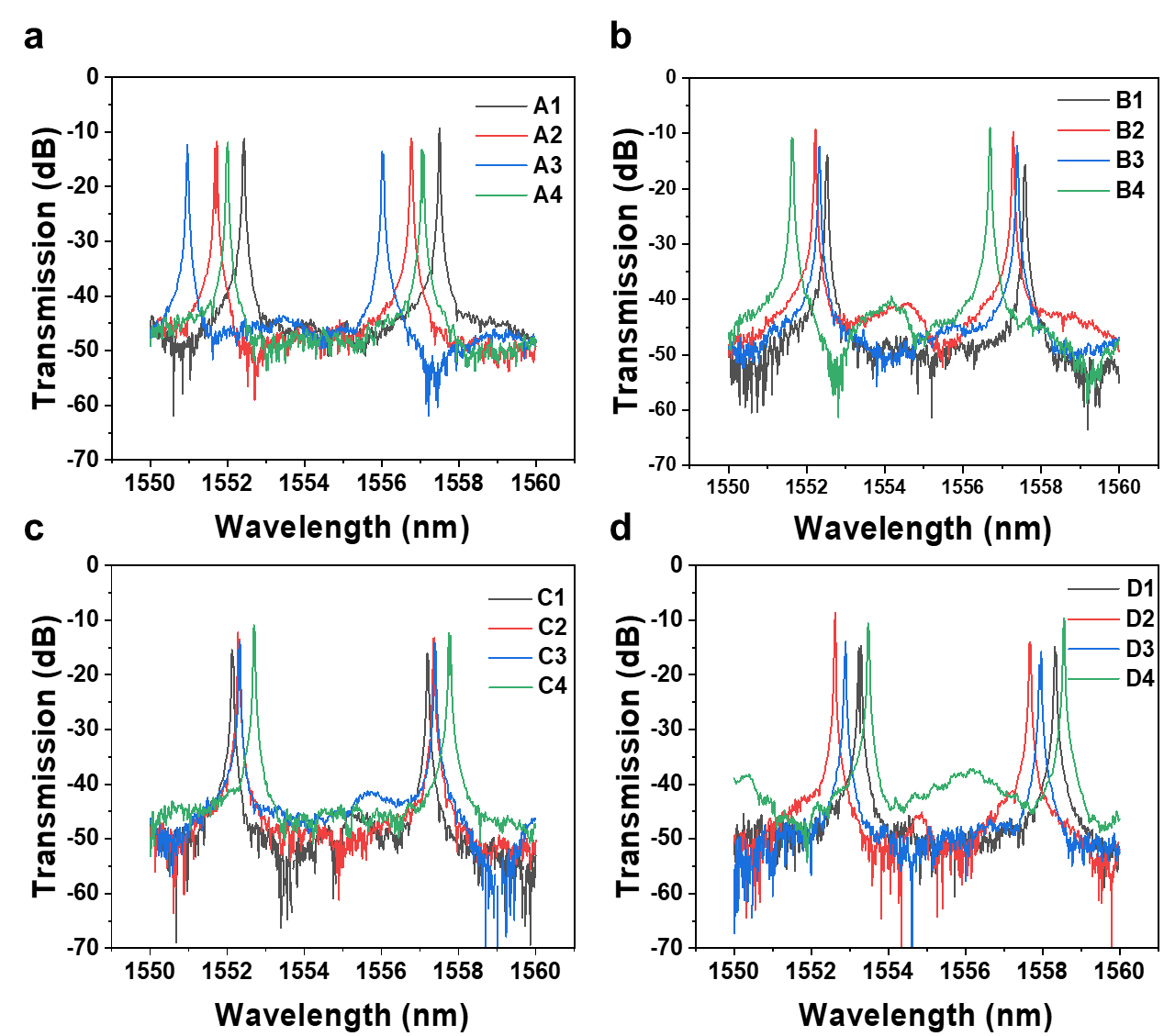


**Figure S6**. The transmission of each MRR. (a) The transmission of type-A MRR. The radius of type-A MRR is 17.99 µm. (b) The transmission of type-B MRR. The radius of type-B MRR is 18.00 µm. (c) The transmission of type-C MRR. The radius of type-C MRR is 18.01 µm. (d) The transmission of type-D MRR. The radius of type-D MRR is 18.02 µm.

Due to fabrication errors, the spectrum of each microring resonator (MRR) varies shown in **Figure S6**. Despite the array consisting of four types of MRRs with different radii (17.99 µm, 18.00 µm, 18.01 µm, and 18.02 µm), there is variation within each type due to fabrication deviations. Since each MRR exhibits a unique spectrum, each has its own lookup table; therefore, a 4x4 array comprises 16 lookup tables.

Regarding the storage of lookup tables, using a 12-bit DAC and a 12-bit ADC requires 2*2^12 bits to store the voltages and weights for a single MRR, which totals 8kB. Consequently, for an N*N MRR array, 8N² kB of storage is needed. However, in convolutional operations where the weights are fixed, there is no need to alter the weights during operation, thereby reducing the demand for high-speed lookup table storage. As a result, the lookup tables can be accommodated in NAND flash or DRAM, rather than the more limited SRAM. Thus, as the array scale increases, the storage of lookup tables will not pose a significant challenge.

## Not**e S5 Variable optic**al attenuator characterizes

**Figure S7.** The transmission rate of a VOA under voltage tuning. This curve represents the normalized W-V mapping of VOA.

## Note S6 Theoretical analysis and fitting of Mach-Zehnder interferometer sensor


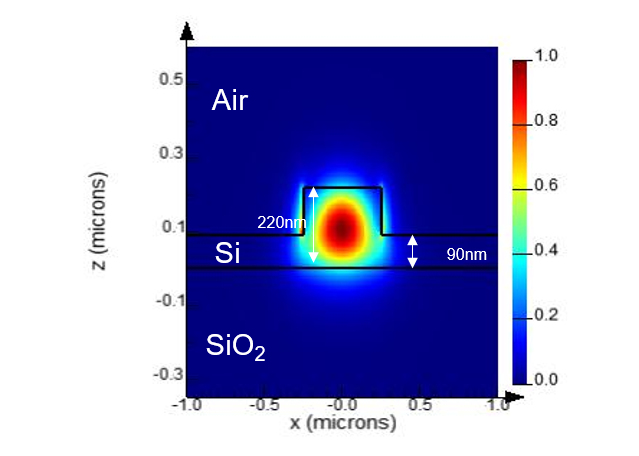


**Figure S8.** The distribution of electric field magnitude in X-Z cross-section.

The distribution of electric field magnitude of the sensing arm of MZI is shown in **Figure S8**. Based on the slab structure, the external confinement factor of 10.07% is extracted by simulation.


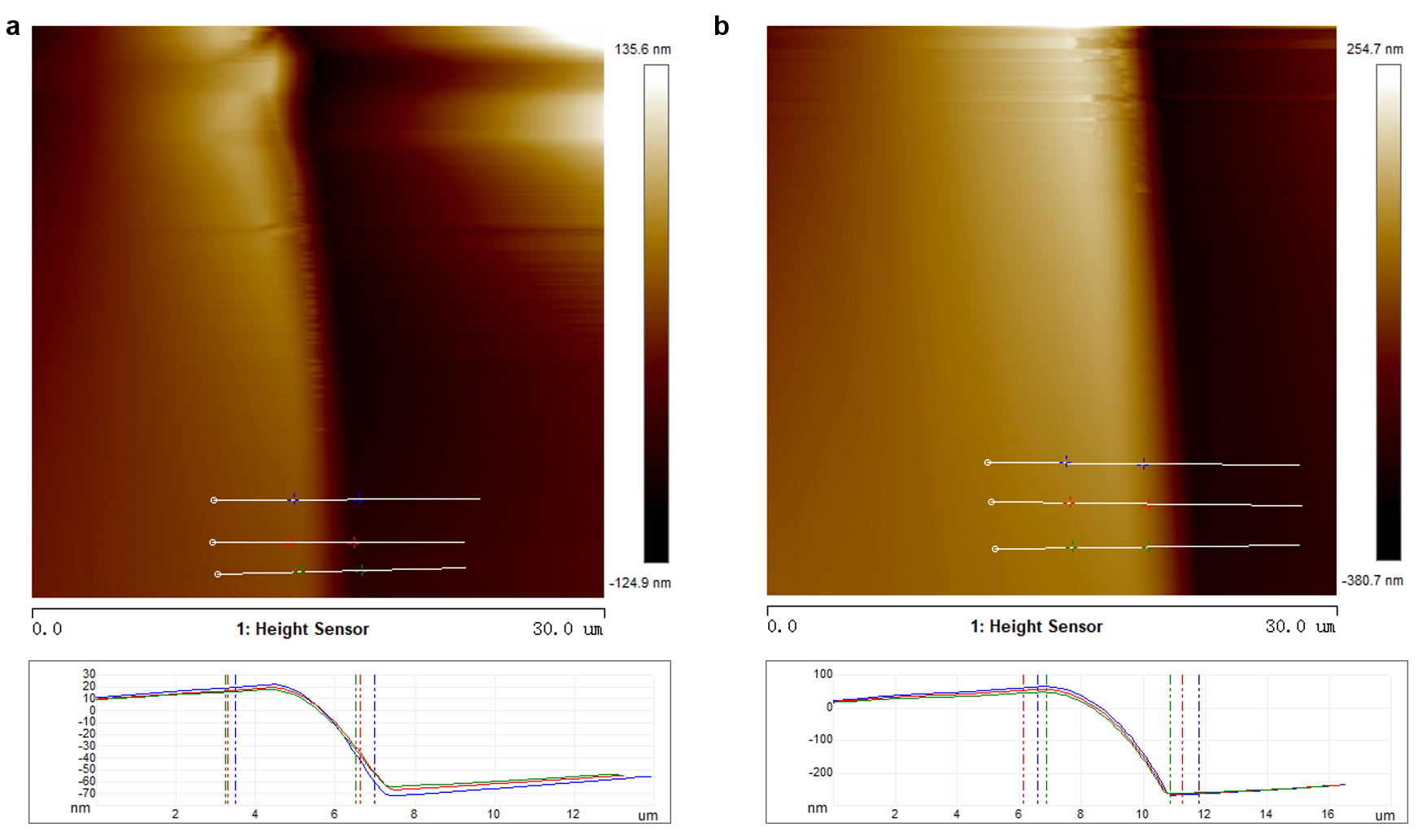


**Figure S9.** AFM characterization of the film thickness.

The protein film thickness is measured by AFM. The **Figure S9a** shows the thickness of $0.2 \mu L$ β-lactoglobulin (BLG) which concentration is $150ng/\mu L$ is about 86.57nm. The **Figure S9b** shows the thickness of $0.2 \mu L$ bovine serum albumin (BSA) which concentration is $150ng/\mu L$ is about 327.40nm. The thickness is calculated by averaging the measurements from 3 sections, which are the dashed line in the figures.


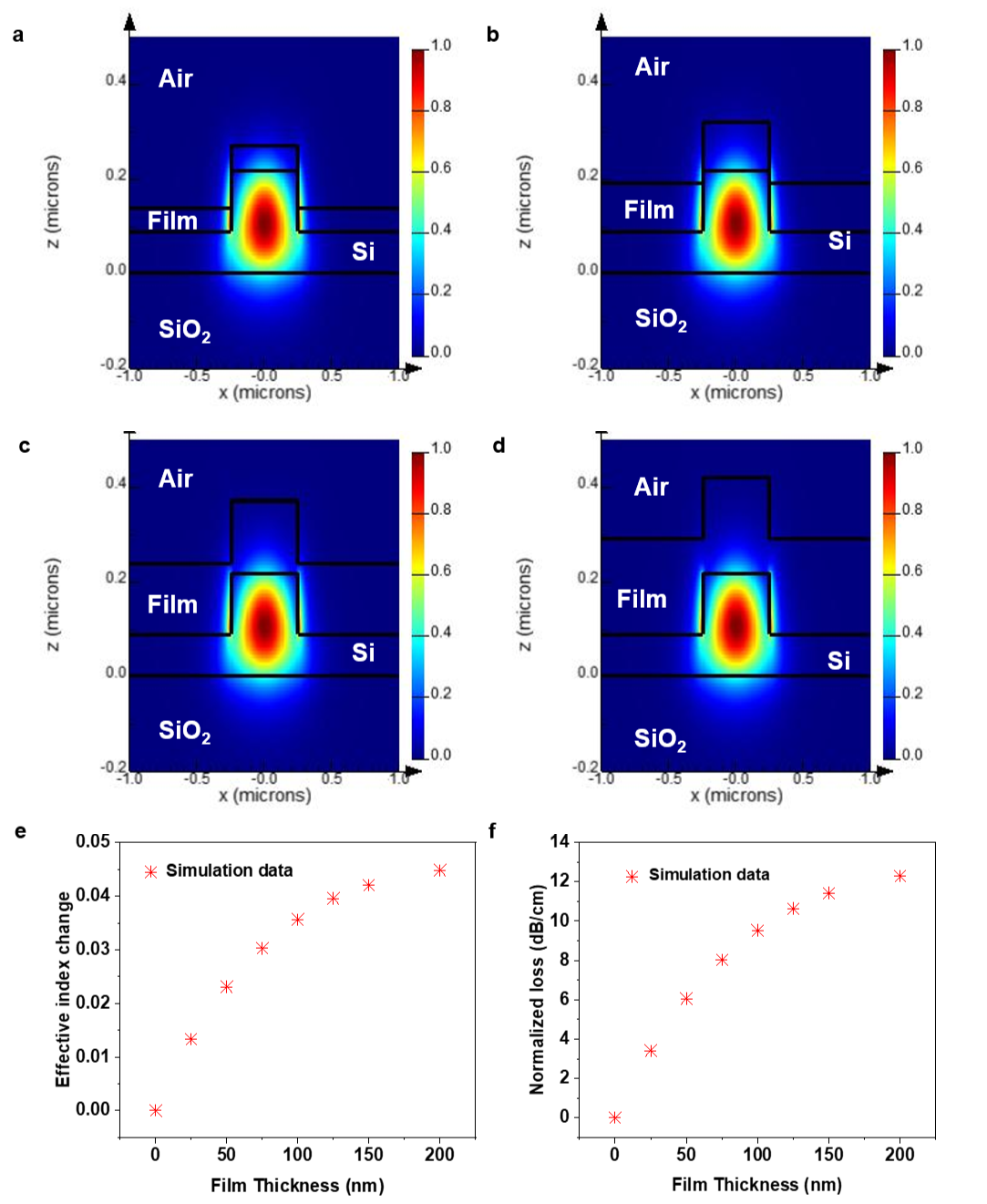


**Figure S10.** (a-d) The distribution of electric field magnitude in X-Z cross-section with the film at the thickness of 50nm, 100nm, 150nm and 200nm. (e-f) The simulation result of effective index changes and normalized loss.

**Figure S10a-d** show the distribution of electric field of the slab waveguide with different film thickness. And **Figure S10e-f** shows their effective index change and loss with different film thickness. The simulation results show that the effective index change, and loss increase with the film thickness. But the film is not the uniform medium in reality.


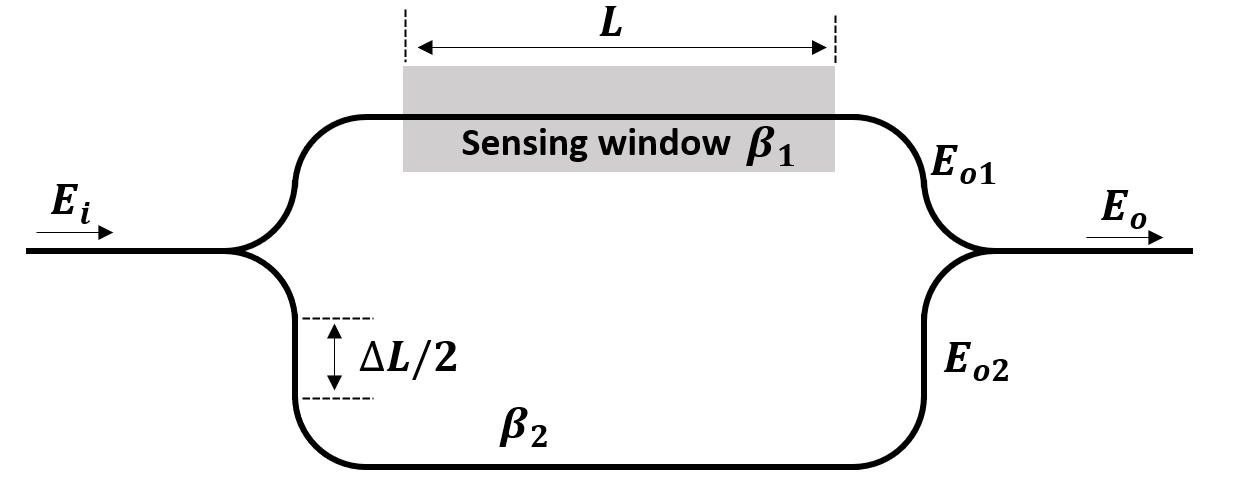


**Figure S11.** Schematic of the imbalanced Mach-Zehnder interferometer (MZI) sensor. $E_{i}$and $E_{o}$ are the electric field for the input and output respectively; $\beta_{1}$is the propagation constants for the waveguide without cladding for sensing which is described by$\beta_{1}=\frac{2\pi n_{sen}}{\lambda}$; $\beta_{2}$ is the propagation constants for the waveguide with cladding, which is for reference arm, $\beta_{2}=\frac{2\pi n_{ref}}{\lambda}$; $\alpha_{1}$, $\alpha_{2}$ is absorption coefficient for the sensing and reference waveguides respectively.

For an imbalanced MZI with length difference $\Delta L$, at the end of two waveguides (at the input to the combiner), the electrical field are described respectively as:

$$\begin{aligned} E_{o1}=\frac{E_{i}}{\sqrt{2}}e^{-i\beta_{1}L-\frac{\alpha_{1}L}{2}}\#\left( Equation S1 \right) \end{aligned}$$

$$\begin{aligned} E_{o2}=\frac{E_{i}}{\sqrt{2}}e^{-i\beta_{2}\left( L+\Delta L \right)-\frac{\alpha_{2}}{2}\left( L+\Delta L \right)}\#\left( Equation S2 \right) \end{aligned}$$

The output of the combiner is:

$E_{o}=\frac{1}{\sqrt{2}}\left( E_{o1}+E_{o2} \right)=$ $\frac{E_{i}}{2}\left[ e^{-i\left( \beta_{1}L \right)-\frac{\alpha_{1}L}{2}}+e^{-i\beta_{2}\left( L+\Delta L \right)-\frac{\alpha_{2}\left( L+\Delta L \right)}{2}} \right]$

$$\begin{aligned} =\frac{E_{i}}{2}e^{-i\left( \beta_{1}L \right)-\frac{\alpha_{1}L}{2}}\left( 1+e^{i\Delta\phi\left( \lambda\right)-\frac{L\left( \alpha_{2}-\alpha_{1} \right)+\alpha_{2}\Delta L}{2}} \right)\#\left( Equation S3 \right) \end{aligned}$$

Where phase difference $\Delta\phi\left( \lambda\right)=\left( \beta_{1}-\beta_{2} \right)L-\beta_{2}\Delta L=\frac{2\pi\lambda}{FSR(\lambda)}$

Here, the power imbalance of the unbalanced MZI sensor is mainly attributed to propagation loss difference due to the differential length $\Delta L$ and sensing arm length $L[1,2]$, defined as:

$$\begin{aligned} Imb\left( magnitude \right)=\frac{\left| E_{o1} \right|^{2}}{\left| E_{o2} \right|^{2}}=e^{\alpha_{2}\Delta L+\left( \alpha_{2}-\alpha_{1} \right)L}\#\left( Equation S4 \right) \end{aligned}$$

For simplicity, we assume that the reference arm losses are negligible $\alpha_{2}=0$. So $Imb$ become:

$$\begin{aligned} Imb\left( magnitude \right)=\frac{\left| E_{o1} \right|^{2}}{\left| E_{o2} \right|^{2}}=e^{{-\alpha}_{1}L}\#\left( Equation S5 \right) \end{aligned}$$

The optical transmission at the output is:

$$\begin{aligned} T\left( \lambda\right)=\frac{\left| E_{o} \right|^{2}}{\left| E_{i} \right|^{2}}=\frac{1}{4}e^{-\alpha_{1}L}\left| 1+e^{i\Delta\phi\left( \lambda\right)-\frac{L\left( \alpha_{2}-\alpha_{1} \right)+\alpha_{2}\Delta L}{2}} \right|^{2} \\ =\frac{1}{4}e^{-\alpha_{1}L}\left[ 1+Imb+2\sqrt{Imb}\cdot\cos\left( \frac{2\pi\lambda}{FSR\left( \lambda\right)} \right) \right]\#\left( Equation S6 \right) \end{aligned}$$

So,

$$\begin{aligned} {T\left( \lambda\right)}_{max}=\frac{1}{4}e^{-\alpha_{1}L}\left[ 1+e^{-\alpha_{1}L}+2e^{\frac{-\alpha_{1}L}{2}} \right]\#\left( Equation S7 \right) \end{aligned}$$

$$\begin{aligned} {T\left( \lambda\right)}_{min}=\frac{1}{4}e^{-\alpha_{1}L}\left[ 1+e^{-\alpha_{1}L}-2e^{\frac{{-\alpha}_{1}L}{2}} \right]\#\left( Equation S8 \right) \end{aligned}$$

For extinction ratio, we take

$$\begin{aligned} ER=10\lg\left( \frac{{T\left( \lambda\right)}_{max}}{{T\left( \lambda\right)}_{min}} \right)=10\lg\left( \frac{1+e^{-\alpha_{1}L}+2e^{\frac{-\alpha_{1}L}{2}}}{1+e^{-\alpha_{1}L}-2e^{\frac{{-\alpha}_{1}L}{2}}} \right)\#\left( Equation S9 \right) \end{aligned}$$

## Note S7 Calculation of limit of detection (LoD) of Mach-Zehnder interferometer sensor

**Figure S12.** The noise floor as a function of the signal intensity.

The noise level as a function of the signal measured in our system is shown. The slope of the linear fitting indicates a laser power fluctuation of 0.149% of the signal, while the intercept indicates a constant photodetector noise of approximately 223.22 μV.

In our experiment for glycerin sensing, the Beer-Lambert’s law is shown below:

$$\begin{aligned} T=\frac{I_{protein}}{I_{nothing}}=\exp\left( -\Delta\alpha\Gamma LC \right)\#\left( Equation S10 \right) \end{aligned}$$

where the $\Delta\alpha$ is the difference in absorption coefficient between protein film and air. Then the equation of calculating the LoD of protein film based on 3-Sigma rule can be expressed as:

$$\begin{aligned} \frac{I_{air}\left[ 1-\exp\left( -\Delta\alpha\Gamma LC_{LoD} \right) \right]}{I_{noise}}=3\#\left( Equation S11 \right) \end{aligned}$$

where the $C_{LoD}$ is the LoD of protein film. For the common cases of noise characterization of absorption-based waveguide sensor, it is normally divided into two cases for analyze^[3–5]^: (i) $I_{noise}=constant$, which mainly comes from the photodetector; (ii) $I_{noise}=kI+C$, the noise floor that mainly origin from laser power fluctuation, is linearly related to the signal intensity. The noise measure result of our sensing platform is shown in **Figure S10**. A clear linear fitting result suggests the noise in our measurement range belongs to the second case, i.e., the noise is proportional to the signal intensity. The typical value of $I_{air}$ (reference signal) in the measurement is ~ 651 mV. Based on the linear fitting equation: $I_{noise}=0.00149I_{signal}+223.22$, This reference signal produces a noise floor of 1.19 mV. The term of $\Delta\alpha\Gamma LC_{LoD}$ in **Equation S10** represents the sensitivity of the sensor and can be extracted from the experiment. Taking the calculated noise floor and $\Delta\alpha\Gamma LC_{LoD}= \frac{-0.0168 dB/(ng/\mu L)}{{log}_{10}e}$ (due to the base-10 logarithm used in absorbance while natural logarithm is used in Beer-Lambert’s law) to **Equation S11**, we can get the $C_{LoD}= 3.84\times{10}^{-3} ng/\mu L$for β-lactoglobulin detection based on the 3-Sigma rule. Use the same way, we can get $C_{LoD}= 2.39\times{10}^{-3} ng/\mu L$for $C_{LoD}= 2.41\times{10}^{-3} ng/\mu L$BSA detection

## Note S8 Comparison of MLP and CNN for spectrum classification

**Table S1 Comparison table for labeling mixtures under different mixing ratios**

| **BSA Concentration**  **(ng/µL)** | **BLG Concentration**  **(ng/µL)** | **Temperature (°C)** | | | | |
| --- | --- | --- | --- | --- | --- | --- |
|  |  | **20** | **22** | **24** | **26** | **28** |
| **5** | **5** | Label 0 | Label 1 | Label 2 | Label 3 | Label 4 |
|  | **20** | Label 5 | Label 6 | Label 7 | Label 8 | Label 9 |
|  | **50** | Label 10 | Label 11 | Label 12 | Label 13 | Label 14 |
| **20** | **5** | Label 15 | Label 16 | Label 17 | Label 18 | Label 19 |
|  | **20** | Label 20 | Label 21 | Label 22 | Label 23 | Label 24 |
|  | **50** | Label 25 | Label 26 | Label 27 | Label 28 | Label 29 |
| **50** | **5** | Label 30 | Label 31 | Label 32 | Label 33 | Label 34 |
|  | **20** | Label 35 | Label 36 | Label 37 | Label 38 | Label 39 |
|  | **50** | Label 40 | Label 41 | Label 42 | Label 43 | Label 44 |


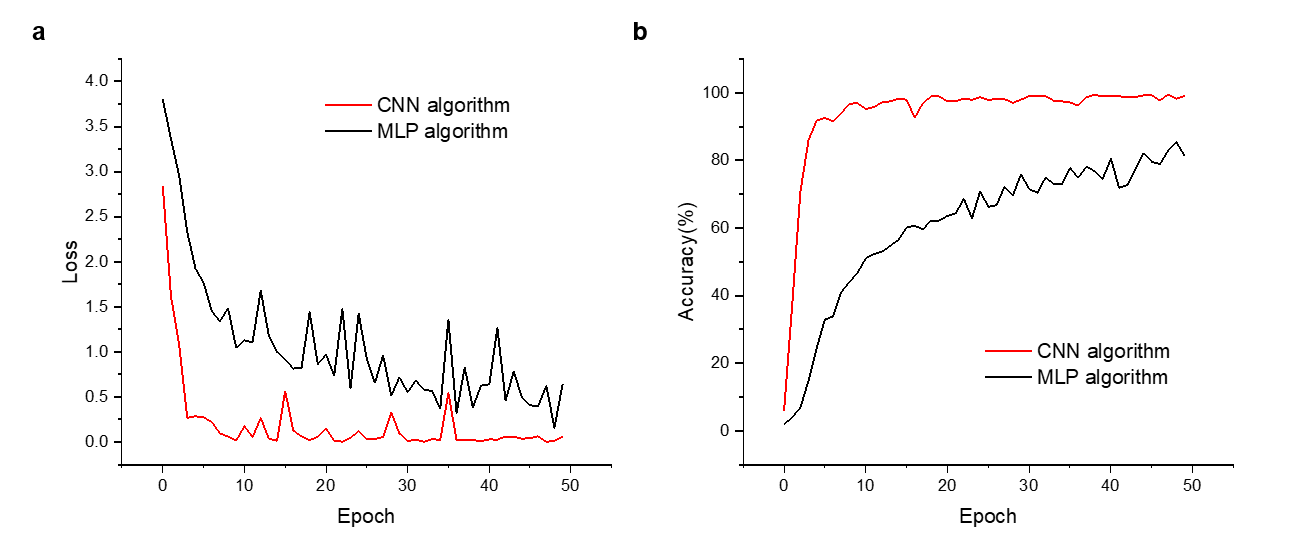


**Figure S13.** (a)Loss and (b)accuracy for mixture classification using different algorithm.

The MLP algorithm and CNN algorithm were utilized for spectrum classification. The results indicate that the CNN algorithm exhibits superior performance in classifying spectra.

## Note S9 The bar charts of testing accuracy on PC


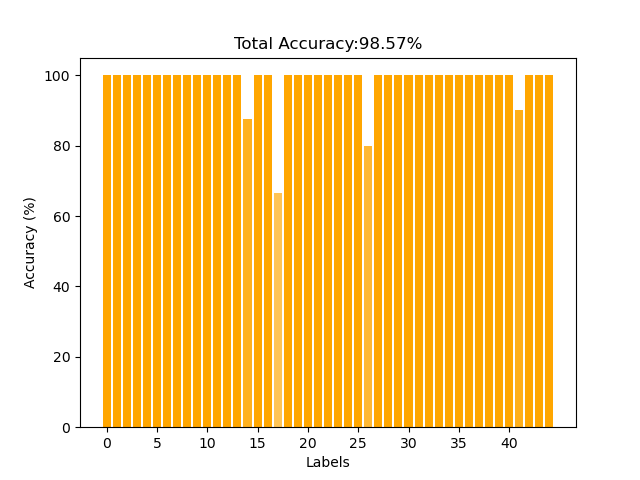


**Figure S14.** The bar charts of testing accuracy in identifying 45 mixtures through PC. The classification accuracy is 98.57%.

## Note S10 The prediction of BSA and BLG


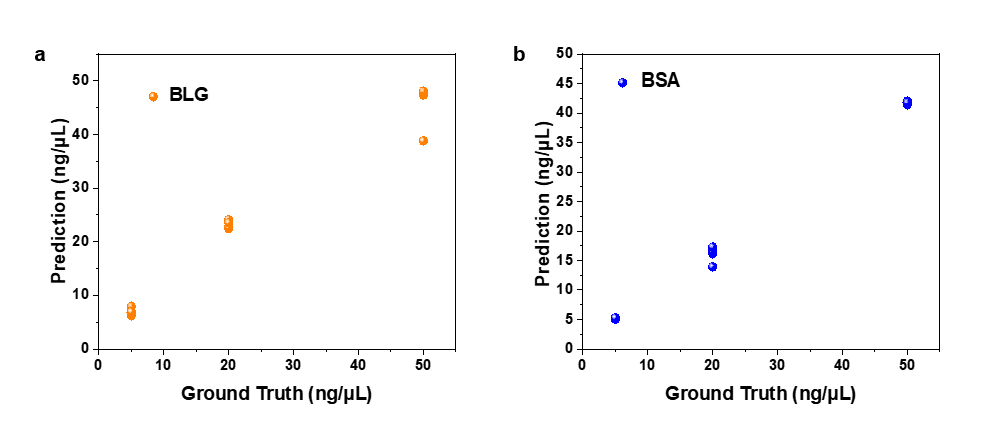


**Figure S15**. Predicted concentration of (a) BLG and (b) BSA at different expected concentrations (ground truth).

## Note S11 Power consumption of whole system estimation

**Table S2. The power consumption of the whole in-sensor computing system**

| Components | Voltage(V) | Current(A) | Power(W) |
| --- | --- | --- | --- |
| Lasers |  |  | $\sim10\times{10}^{-3}\times4=0.04$ |
| On-ring heaters | $\approx0.5$ | $1\times{10}^{-3}$ | $5\times{10}^{-4}\times16=8\times{10}^{-3}$ |
| VOAs | $\approx3$ | $1.4\times{10}^{-3}$ | $4.2\times{10}^{-4}\times4=1.68\times{10}^{-3}$ |
| TEC | $\approx0.5$ | $\approx0.12$ | 0.06 |
| Total power consumption |  |  | 0.11 |

The throughput of photonic computing hardware can be calculated by Eq. (S12)^[6]^:

$$\begin{aligned} T=2m\times N^{2}\times f OPS\#\left( Equation S12 \right) \end{aligned}$$

where *T* is the throughput in units of OPS (operations per second), *m* is the number of layers implemented by photonic computing hardware, *N* is the size of on-chip weight bank, and *f* is the detection rate of the PDs. Since the in-sensor computing system naturally performs multiplication and accumulation (MAC) operations, and each MAC operation consists of one multiplication operation and one accumulation operation, one MAC operation corresponds to two operations. At 5 GHz photonic detection rate (THORLABS, DET08CFC/M), our in-sensor computing system proof-of-concept chip (N^2^ =4×4) have throughputs of 0.32 TOPS ($2\times2\times4\times4\times5G$). Therefore, the power efficiency of the in-sensor computing system is $2.9 TOPS/W$.

## Note S12 Comparison with other sensing system.

Unimodal photonic sensors are susceptible to temperature variations, whereas multimodal photonic sensors are not. Moreover, separated photonic sensors require additional lasers and photodetectors to acquire spectroscopy data, leading to higher measurement costs and larger physical footprint. *The size of single MZI sensor is* $150\mu m\times2300\mu m$*.* The table below provides further details.

Table S3 Comparison with separated unimodal sensors

| Kinds | Three photonic separated unimodal sensors. | multimodal photonic sensor |
| --- | --- | --- |
| Footprint | 3*0.345mm^2^=1.035 mm^2^ | 0.345=0.345 mm^2^ |
| Laser used | 3 tunable lasers | 1 tunable laser |
| Photodetector used | 3 photodetectors | 1 photodetector |
| Power consumption | 3*(10+16.5) = 79.5mW | 10+16.5=26.5 mW |

To ensure that the three separate sensors acquire spectroscopy data simultaneously, the tunable lasers must operate at the same wavelength at the same time. Otherwise, the measurement time for multimodal sensing will increase.

Our machine learning model requires approximately 1 million multiply-accumulate operations (MAC). The calculation details are shown below.

For a convolution layer:

$$\begin{aligned} MAC=output_{channels}\times output_{height}\times output_{width}\times kernel_{height} \\ \times hernwl_{width}\times input_{channels}\#\left( Equation S12 \right) \end{aligned}$$

For a Fully connected layer:

$$\begin{aligned} MAC=\mathrm{input}_{\mathrm{size}}\boldsymbol{\times}\mathrm{output}_{\mathrm{size}}\#\left( Equation S13 \right) \end{aligned}$$

For comparison, a Tesla V100 GPU delivers 30 trillion operations per second (30 TOPS) with a power consumption of 300W, resulting in an efficiency of 0.1 TOPS/W.^[7]^ In contrast, electrical processing for machine learning consumes around 10 µW. Some other approaches implement linear and nonlinear functions optically,^[8,9]^ ideally eliminating the need for post-electrical processing. Photonic processing consumes only 0.3 µW. Therefore, such multimodal in-sensor computing system ideally is 30 times more power-efficient than conventional GPU-based systems.

Reference

[1] P. Edinger, A. Y. Takabayashi, C. Errando-Herranz, U. Khan, H. Sattari, P. Verheyen, W. Bogaerts, N. Quack, K. B. Gylfason, *Opt Lett* **2021**, *46*, 5671.

[2] S. Xu, Z. Ren, B. Dong, J. Zhou, W. Liu, C. Lee, *Adv Opt Mater* **2023**, *11*, DOI: 10.1002/adom.202202228.

[3] J. Zhou, Z. Zhang, B. Dong, Z. Ren, W. Liu, C. Lee, *ACS Nano* **2023**, *17*, 711.

[4] Y. Ma, Y. Chang, B. Dong, J. Wei, W. Liu, C. Lee, *ACS Nano* **2021**, *15*, 10084.

[5] W. Liu, Y. Ma, Y. Chang, B. Dong, J. Wei, Z. Ren, C. Lee, *Nanophotonics* **2021**, *10*, 1861.

[6] Y. Shen, N. C. Harris, S. Skirlo, M. Prabhu, T. Baehr-Jones, M. Hochberg, X. Sun, S. Zhao, H. Larochelle, D. Englund, M. Soljačić, *Nat Photonics* **2017**, *11*, 441.

[7] S. Ambrogio, P. Narayanan, H. Tsai, R. M. Shelby, I. Boybat, C. di Nolfo, S. Sidler, M. Giordano, M. Bodini, N. C. P. Farinha, B. Killeen, C. Cheng, Y. Jaoudi, G. W. Burr, *Nature* **2018**, *558*, 60.

[8] Y. Shi, J. Ren, G. Chen, W. Liu, C. Jin, X. Guo, Y. Yu, X. Zhang, *Nat Commun* **2022**, *13*, 6048.

[9] F. Ashtiani, A. J. Geers, F. Aflatouni, *Nature* **2022**, *606*, 501.
